# SOMWeb: A Semantic Web-Based System for Supporting Collaboration of Distributed Medical Communities of Practice

**DOI:** 10.2196/jmir.1059

**Published:** 2008-08-24

**Authors:** Göran Falkman, Marie Gustafsson, Mats Jontell, Olof Torgersson

**Affiliations:** ^3^Department of Computer Science and EngineeringUniversity of Gothenburg / Chalmers University of TechnologyGothenburgSweden; ^2^Institute of OdontologySahlgrenska AcademyUniversity of GothenburgGothenburgSweden; ^1^School of Humanities and InformaticsUniversity of SkövdeSkövdeSweden

**Keywords:** Dental informatics, medical informatics applications, communications applications, community networks, group and organization interfaces, interdisciplinary communication, Internet, knowledge bases, online information services, user/machine systems

## Abstract

**Background:**

Information technology (IT) support for remote collaboration of geographically distributed communities of practice (CoP) in health care must deal with a number of sociotechnical aspects of communication within the community. In the mid-1990s, participants of the Swedish Oral Medicine Network (SOMNet) began discussing patient cases in telephone conferences. The cases were distributed prior to the conferences using PowerPoint and email. For the technical support of online CoP, Semantic Web technologies can potentially fulfill needs of knowledge reuse, data exchange, and reasoning based on ontologies. However, more research is needed on the use of Semantic Web technologies in practice.

**Objectives:**

The objectives of this research were to (1) study the communication of distributed health care professionals in oral medicine; (2) apply Semantic Web technologies to describe community data and oral medicine knowledge; (3) develop an online CoP, Swedish Oral Medicine Web (SOMWeb), centered on user-contributed case descriptions and meetings; and (4) evaluate SOMWeb and study how work practices change with IT support.

**Methods:**

Based on Java, and using the Web Ontology Language and Resource Description Framework for handling community data and oral medicine knowledge, SOMWeb was developed using a user-centered and iterative approach. For studying the work practices and evaluating the system, a mixed-method approach of interviews, observations, and a questionnaire was used.

**Results:**

By May 2008, there were 90 registered users of SOMWeb, 93 cases had been added, and 18 meetings had utilized the system. The introduction of SOMWeb has improved the structure of meetings and their discussions, and a tenfold increase in the number of participants has been observed. Users submit cases to seek advice on diagnosis or treatment, to show an unusual case, or to create discussion. Identified barriers to submitting cases are lack of time, concern about whether the case is interesting enough, and showing gaps in one’s own knowledge. Three levels of member participation are discernable: a core group that contributes most cases and most meeting feedback; an active group that participates often but only sometimes contribute cases and feedback; and a large peripheral group that seldom or never contribute cases or feedback.

**Conclusions:**

SOMWeb is beneficial for individual clinicians as well as for the SOMNet community. The system provides an opportunity for its members to share both high quality clinical practice knowledge and external evidence related to complex oral medicine cases. The foundation in Semantic Web technologies enables formalization and structuring of case data that can be used for further reasoning and research. Main success factors are the long history of collaboration between different disciplines, the user-centered development approach, the existence of a “champion” within the field, and nontechnical community aspects already being in place.

## Introduction

### Motivation

Oral medicine is a subdiscipline of dentistry concerned with diseases related to the oral and paraoral structures, including the principles of medicine related to the mouth as well as diseases specific to the orofacial tissues and oral manifestations of systemic diseases. Oral medicine is a quite small and growing subdiscipline. It often deals with disorders of low prevalence, and to enhance the knowledge, gathering of clinical data from large geographic areas is needed. This means that cooperation between geographically distributed clinics is vital for providing a means of consultation and learning for a broader audience and for collecting diverse and numerous cases for further analysis and teaching.

The Swedish Oral Medicine Network (SOMNet) promotes knowledge sharing and dissemination between clinicians and researchers in oral medicine in Sweden. The central activity of SOMNet is regularly held distributed meetings focused on case discussions. These meetings are conducted using telephone conferences. The cases to be discussed are distributed among SOMNet members before the meetings. Before the introduction of the system presented here, cases were presented as PowerPoint presentations, which were emailed prior to the meetings. As the number of participants grew, emailing was abandoned in favor of an online repository of presentations converted to HTML. Several drawbacks with this solution were identified, such as the lack of connection between follow-up presentations and the original case, no common structure for information entered, and no support for searching and browsing the cases. This led to considerations of developing an online solution more tailored to the needs of SOMNet that should complement the speech-based interaction with Internet-supported management of structured case descriptions and images.

Modern information technology (IT) in general and the Internet in particular provide the technical infrastructure for supporting interdisciplinary clinical teamwork [1]. Benefits include the possibility of distance consultation and accessing remote expertise [2], sharing clinical data and imagery, dissemination of information and knowledge through broadcasted seminars and online courses [3], and distributed virtual work places [4]. Internet and IT are of course prevalent within eHealth. However, so far, most work has been on telemedicine and consumer health informatics, and the utility of eHealth systems to promote clinical teamwork and collaboration has received little attention [1,3]. Even more so, given the current focus on evidence-based medicine [5], the need for research on how daily clinical practice can be used as a basis for further scientific activities within a distributed medical community is eminent [3,6].

The design, development, and adoption of IT-supported tools for clinical activities within distributed medical communities is a sociotechnical problem [7,8], requiring more research on the communication and knowledge processes used by community members in everyday practice and research [1,9], social and behavioral factors influencing the adoption and use of tools [3,10], and research on how to take full advantage of the capacities of the Internet and the computer as essentially new media for conducting clinical practice and medical research [11-13].

One way to promote the knowledge sharing and dissemination is to provide IT support for communities of practice (CoP). A community of practice is a group of people who share “a concern, a set of problems, or a passion about a topic, and who deepen their knowledge and expertise in this area by interacting on an ongoing basis” [14]. Of special interest is the possibility of using the Internet to support virtual CoP, where members are geographically dispersed and where face-to-face meetings are rare. A virtual CoP has at its disposal both traditional media, such as telephone and telephone conferences, as well as more recent technological tools, such as email, databases, websites, and online meeting places [15]. Internet-based CoP can play an important role in the externalization of tacit knowledge of the individual (eg, clinical practice knowledge) into explicit and diffused knowledge (eg, evidence, protocols, or clinical guidelines) [16].

Semantic Web technologies have been put forward as enablers of Internet-based tools supporting knowledge-intensive tasks [15,17]. A key component of the Semantic Web is the representation of knowledge in a computer-processable manner, in the form of ontologies [18]. However, despite much effort, the adoption of ontologies within the medical domain has turned out to be more problematic and slower than many had hoped [19], and there is a need for successful examples showing how Semantic Web technologies can be put to use.

In terms of online health information systems, interactivity and end-user control are instrumental in creating “a sense of mutuality, connection, common ground, and shared understanding, and, ultimately, participation in medical decision making” and for enhancing “elaboration and learning of complicated concepts that require understanding linkages between concepts” [13]. Poor interface design and tools not being well adapted to the tasks at hand and not seamlessly integrated into workflows are causes of failure of medical collaboratories [3]. Development of tools in collaboration between user and developer, where prototypes are tested in the daily clinical activity, has been identified as a key success factor in the development and adoption of health information systems [20,21]. Tools should be simple and adaptable to the individual user’s preferences and needs [22].

### Aims and Objectives

The aims of the presented research are to acquire (1) a better understanding of how clinicians in oral medicine communicate and collaborate, (2) more knowledge about the design, development, and adoption of Internet-based tools for distributed clinical practice, learning, and research, and (3) experience in applying Semantic Web technologies to realistic examples.

These aims are reached by fulfilling the following objectives: (1) study the communication of distributed health care professionals in oral medicine, within the framework of CoP; (2) apply Semantic Web technologies to describe community data and oral medicine knowledge; (3) design, develop, and adopt an online CoP of oral medicine (SOMWeb) centered around user-contributed case descriptions and meetings, which, in order to increase user acceptance, should be user-centered and user-controllable and should be based on iterative testing and validation of the computer support in daily clinical work; (4) evaluate the online CoP and study how communication and work practices change with IT support.

## Methods

### Study Context

SOMNet was initiated in the early 1990s to share oral medicine knowledge and make possible consultations in a fairly small discipline where clinics are geographically dispersed. SOMNet can also be seen as an instrument for continuing education, for harmonizing terminology, and for building a database of interesting cases in oral medicine. The participants are distributed throughout Sweden, in clinics at hospitals, primary care facilities, and in private practice. The members of SOMNet are mainly dentists with an interest in oral medicine. Some, but not all, of the participants have been certified by the Swedish Oral Medicine Society (SOMS). Among the participants are general practitioner dentists, hospital dentists, specialists in jaw surgery and oral medicine, professors, and some oral pathologists.

SOMNet’s members have had access to different generations of IT support for their teleconferences. To identify drawbacks with the previously described PowerPoint-based solution and come up with requirements for a new system, we observed meetings, interviewed several members, and used an online questionnaire. The observations were done at the clinic of oral medicine, at the Sahlgrenska Academy in Gothenburg. Among the identified needs were the ability to add cases in a more structured manner, to assign cases to meetings both for initial consultation and for follow-up, to view cases allocated to a given meeting, and to search and browse the case repository.

The SOMWeb system was constructed to provide the above functionality. SOMWeb was developed in cooperation with clinicians starting in spring 2005 and was introduced to all SOMNet members in May 2006. Our primary contact is the clinic for oral medicine at Sahlgrenska Academy in Gothenburg. This is a continuation of more than 10 years of collaboration during which a suite of software, called MedView, has been developed to support the oral medicine clinicians [23].

In SOMWeb, no information is available that will reveal the identity of the individuals in the presented cases. The case data that each presenter has to provide do not contain personal information, except for age, gender, and ethnic background. *En face* images are prohibited, and intraoral images will not disclose identity. All members of SOMNet have signed a professional secrecy agreement as part of their clinical assignment. In addition, all users need an individual username and password in order to access the system.

### Methods of System and Ontology Development

#### System Development

In order enhance user acceptance and system usability, SOMWeb was developed iteratively, following a user-centered design approach, which means that already from the start, a select group of users were involved in the design process [20]. The users took part in the establishment of initial requirements and have continuously provided feedback on developed prototypes.

**Figure 1:**
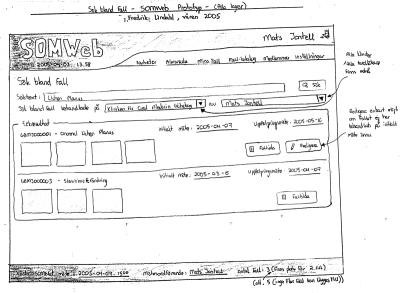
A paper prototype of SOMWeb that shows a text-based search function, restricted to a specified clinic and clinician, which results in a list of cases with associated photos shown as thumbnails, with the option of viewing and editing case data

**Figure 2:**
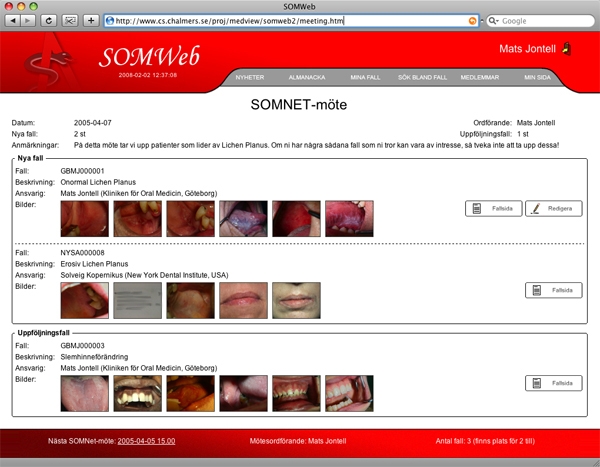
The first HTML prototype of SOMWeb showing a meeting page with new cases to be discussed for the first time and cases that are follow-ups from previous meetings, with photos for each case displayed as a row of thumbnails

As a first step toward replacing the old method of distributing case presentations, a simple Web page was created where submitted cases were made available to members of SOMNet. In parallel, paper prototypes were developed as they are an efficient means to provide initial presentation of a system to users (see Figure 1 for an example). Based on discussions with the user group, an interactive HTML prototype was developed. This prototype, while not containing any real functionality, was fully testable and provided the opportunity to try out what it would be like to work with the system (Figure 2).

The presentation of the prototypes led to deep and lengthy discussions with the user group concerning the exact details of what should be included in the system, how cases should be presented and entered, how follow-up cases should be handled, and so on. Once initial consensus was reached based on the prototypes, development of the first version of the system began. According to the iterative development method, only the basic functionality for adding cases and managing these at meetings was implemented at first. In a later iteration, secondary features like email messaging and a discussion forum were added to the system.

The design rationales are to provide simplicity of interaction and a clean and esthetically attractive user interface design. This was to avoid often-reported problems with medical information systems not having compelling and useful interfaces for the user [24,25]. It was also our experience from previous work that the IT-maturity among clinicians is not very high. To ensure simplicity, only basic functionality is initially available. To have full access to all parts of the system, the users must make an active choice by changing their individual preferences.

From a technical point of view, we have used object-oriented software development methods, such as the Unified Process (UP/RUP), and the Unified Modeling Language according to established design patterns. SOMWeb is built on the Apache Struts Model-2 Web application framework. In Model-2 frameworks, a variation of the classic model-view-controller design paradigm, Java servlets execute business logic with server pages handling the presentation.

#### Ontology Development

Ontologies are used in SOMWeb to represent oral medicine templates and knowledge, as well as to represent community models and data. The Web Ontology Language  (OWL) and Resource Description Framework (RDF), which became a World Wide Web Consortium (W3C) recommendation in February 2004, are used. The knowledge representation and content of MedView are taken as starting points in the design of the oral medicine ontologies of SOMWeb. The OWL ontologies were automatically generated from the previous representation, after extensive work in identifying appropriate corresponding OWL constructs. Identifying external sources for reusing medical knowledge was attempted, but none was found with the appropriate focus. Ontology elements related to community aspects were identified through the iterative modeling and development work described above. For reading and writing OWL and RDF from Java, the Jena programming API was used.

### Methods for Studying Community Collaboration and System Evaluation

Our study of SOMNet’s collaboration and the use of SOMWeb include interviews with participants, observations of teleconference meetings, and an online questionnaire.

The interviews were intended to provide a greater understanding of how SOMWeb is used and how it has affected SOMNet’s meetings and the members’ knowledge use and to identify processes that are part of SOMNet functioning as a distributed CoP. A semistructured interview format was chosen in order to have the flexibility to adapt the interview to the issues brought up by the interviewee. Interviews were thus guided by a semistructured interview guide, which included sections about submitting cases, meeting participation and preparation, knowledge needs and benefits of SOMNet, and use of the SOMWeb system outside of meetings. Questions regarding the addition of cases asked about how the interviewee decides to add a case, how he or she gathers information about the case, and opinions about the form used for entering cases. Regarding meetings, questions asked about how the meetings had changed since using PowerPoint, when the system works well and when it does not work during meetings, how often the interviewee participates and how he or she usually prepares for meetings. Regarding knowledge needs, interviewees were asked about their own benefit in participating in SOMNet, how they perceived the benefit of others, what kind of cases they thought SOMNet should include, and if there was an example of when SOMNet has helped solve a case that might not have been resolved otherwise. Interviewees were also prompted for whether there was anything they wanted to discuss that was not brought up in the questions. The questions were not always brought up in the same order.

We interviewed nine members of SOMNet. Five were interviewed individually, and two interviews were carried out with two members at a time. The first interview was carried out in November 2007 and the last in March 2008. Each interview lasted between 35 and 85 minutes. Three of the interviewees have been members more or less from the start, three have been members for at least 4 years, and three have joined more recently. Two of the respondents are oral pathologists and do not see patients themselves. The other seven all work at hospitals, and two of these have a research background.

Interviews were recorded and transcribed. The questions for the interview were used as initial themes for coding the interviews, but matter that came up spontaneously during interviews was also included in the coding. The interviewer carried out all coding, first by hand on printed transcripts and then by collecting responses from interviewees in a spreadsheet. This compilation was used to compare and count interviewee opinions on different themes. However, due to the qualitative nature of the study and the open-ended responses, a deeper quantitative analysis is not appropriate.

Observations are carried out by sitting at one of the clinics during a telephone conference. The purposes of the observations were to elucidate how cases are presented during teleconference meetings, how clinicians behave locally during these meetings, and how the SOMWeb system is used locally during meetings. The same person did all observations. Notes were taken on both what was said in the telephone conference as a whole and on what the participants said and did locally. One meeting was also recorded and transcribed. In analyzing the data, descriptions of the meeting procedure and case presentations were generalized from notes. Ten meetings were observed: six at the clinic for oral medicine at the Sahlgrenska Academy in Gothenburg and four at four other clinics in Sweden. The first meeting was observed in February 2007 and the last in April 2008.

The online questionnaire contained both open-ended and closed-ended questions. In the first category were questions about reasons for participating and choosing to add cases; in the latter were questions for comparing the SOMWeb system to the PowerPoint-based approach along several facets, such as submitting cases and viewing old cases, where a scale of better, neutral, or worse was used. The questionnaire was made available for about one month in the spring of 2007. Requests for completing the questionnaire were made at a telephone conference, on the news page of the system, and by email to around 60 members, the total number of members at the time. In total, 24 members completed the questionnaire.

Since these observations were carried out by one of the developers of the SOMWeb system, there could be bias both in what the developer observes and in the behavior of the users when observed. This also applies for the interviews. Brender [26] describes pitfalls and biases related to the use of questionnaires and interviews, including psychological factors leading to unwillingness to answer questions due to factors such as prestige, differences between personal opinions and the official account, and mood at the time of responding.

## Results

The results include characteristics of collaboration as studied through the use of SOMWeb, the SOMWeb system, and evaluation aspects pertaining more directly to the system.

### Communication and Collaboration Within SOMNet

#### Meeting Structure and Activities

The SOMNet distance consultations are held once a month (five times in the spring and four times in the fall) by telephone conference. The time scheduled for each meeting is one hour, during which three to six cases are brought up for the first time, and up to three are presented as follow-up cases. A chairperson leads the meeting, for example, by providing transitions between case presentations and by leading and summing up discussions. When presenting a case, the presenter often tells the story of his or her meetings with the patient, treatments tried, and results of these treatments. After, and sometimes during, this short presentation, the other participants ask questions for clarification. Depending on the kind of case presented and what purpose the clinician had for wanting to discuss it, the participants will start suggesting possible diagnoses and treatments. Similar cases or general treatment strategies will sometimes accompany the suggestions. A more broad discussion may ensue, for example, about reported side effects for medications or whether a certain treatment is suitable in general. The chairperson usually starts summarizing when several options have been put forth, and suggestions are given to the presenter. Few participants, apart from the chairperson, took notes at the meetings that were observed.

SOMNet has a lot of experience with teleconferences, and there is a flow in the conversation even though participants cannot see each other. Most participants indicate who is speaking before giving their comment. However, if somebody chooses to have a small local discussion, the flow in conversation is quicker and more interactive.

As of May 2008, 10 to 15 clinics join each meeting. At each of these, there are between one and 10 participants, with an average of three. Where the participants at a local clinic congregate for a teleconference meeting depends on how many participants there are. If only a small number, then they usually sit in front of a computer in an office. If there are more than two or three participants, then usually a meeting room with a projector is used.

#### Meeting Preparations

All interviewees replied that they usually go through the cases before the meeting, either the same day or the night before. While doing this they try to form their own opinion of the cases and find that the benefit of participation increases with this preparation. It was also indicated that there is an obligation toward the case submitter to look at the case before the meeting. The designated chairperson makes a more thorough review of the meeting’s cases and tries to find relevant research literature. Some also add private comments to cases.

A problem brought up is that the cases are submitted too late, which makes it difficult for the members and especially the chairperson to prepare.

#### Purposes for Presenting Cases

Interview responses and observations indicated mainly three purposes for presenting cases: cases where advice regarding diagnosis or treatment is wanted, cases of an unusual character, and cases where the presenter wants to raise an issue for discussion. The advice seeking is most common, but the presenter may have several reasons for bringing a case to the meeting. Examples of recurring discussions are how to monitor patients with precancerous disorders and the reporting of medication side effects.

#### Individual Member and Community Benefits

The value of SOMNet for the majority of participants is access to external expertise and, in the end, better care for patients, in addition to a means of continuing education. Interviewees brought up concrete examples of when they benefited from diagnosis and treatment discussions at meetings, both for cases they presented themselves and from discussions of others’ cases. For example, one respondent described how a special kind of plastic guard described at the meeting had been constructed and used with good results. Another respondent reported how the same technique had been related to, and later applied by, a dentist at a nonparticipating clinic. A third respondent vividly retold the lengthy discussion generated by a difficult case where the symptoms could be construed to have three different causes. Members that are more senior find SOMNet valuable for getting references and comparative material, while maintaining competency and enthusiasm. Meanwhile, the pathologists, with no patients of their own, bring up the social aspects SOMNet provides, in addition to a more complete clinical picture of patients than they usually get. Members at teaching institutions have also included participation in SOMNet as a part of the curricula of some of their courses.

#### Identified Barriers and Issues

The interviewees often raised the issue of a lack of time due to a heavy load of patients or teaching. One interviewee brought up the differences that he feels exist between his work place, where oral medicine is a small part of the overall activities and where there is no research connection, and research institutions with a specialty in oral medicine. He found that it was not possible to set aside office hours to search for and read relevant literature. Fortunately, there were no problems in setting aside an hour for the meeting.

While interviewees at research institutions and some hospitals had access to online articles, smaller hospitals and general practitioners often lacked such access. Interviewees who mainly see patients state that they do not use literature as much as they would like with, again, time being the main barrier. Two interviewees indicated that reading articles was mainly done outside of work hours.

Another matter discussed in interviews was how the participation of smaller clinics can be increased, regarding both contributing cases and participating verbally in the meetings. Connected with this is how the concern about exposing gaps in one’s knowledge can be alleviated. This is also related to what kinds of cases are submitted by the more active case presenters, and some replies to the questionnaire stated that they had considered entering a case but came to the conclusion that it was not “advanced enough.” When this was brought up in interviews with the more active participants, several said that meeting discussions around what appeared to be straightforward cases to them usually turned into very interesting discussions, or as one of the most senior members said, “There isn’t one case that is not interesting enough.”

### The SOMWeb System for Community Collaboration

#### SOMWeb Community Functionality

**Figure 3:**
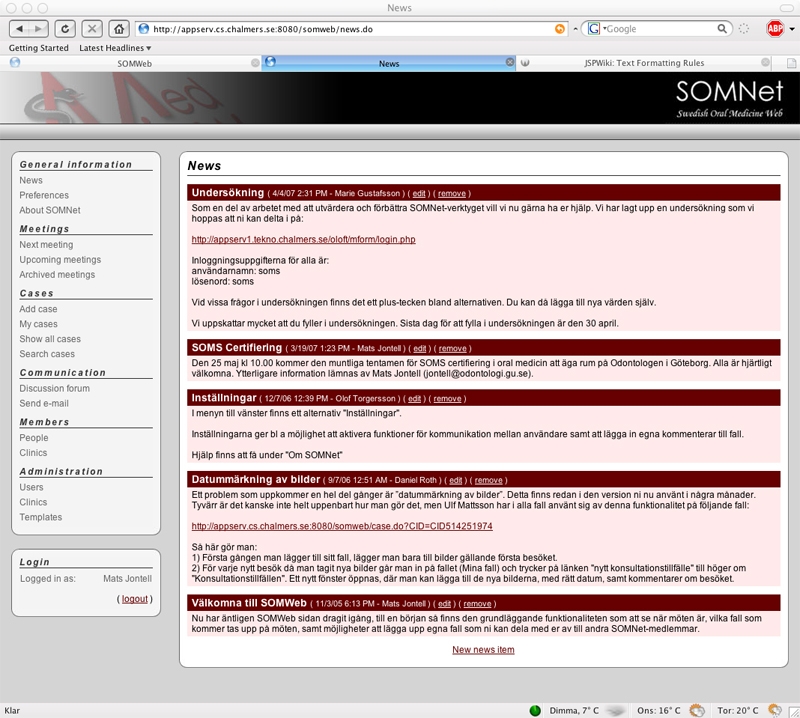
News page with information for all members

The first page a member sees after logging in is a news page (Figure 3), where administrative users put information relevant to all members. Navigation in SOMWeb is done mainly through a menu on the left, with subheadings for main functionality: meetings, cases, communication, and members.

**Figure 4:**
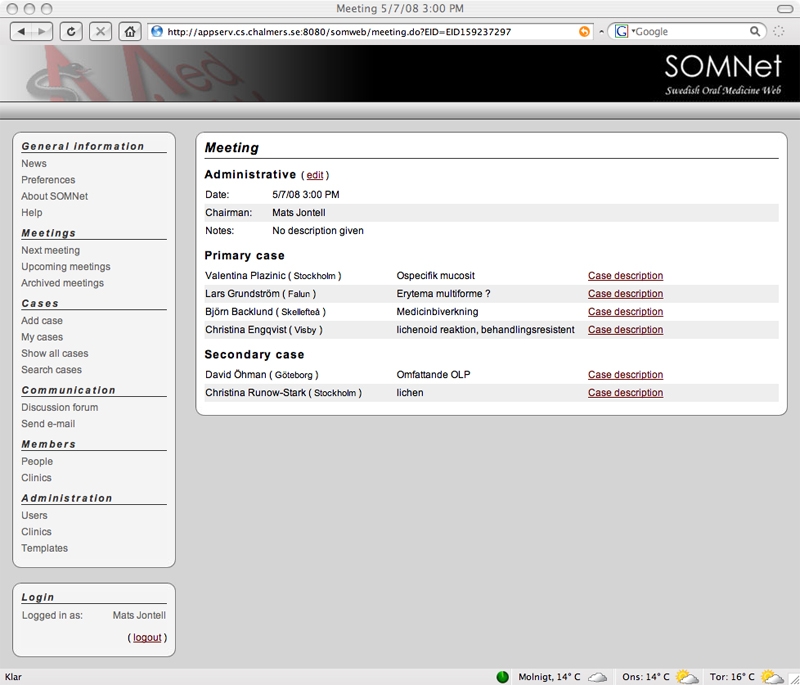
Meeting page with date, chairperson, and list of cases added for discussion at the meeting

Under the meetings subheading, there is a link to the current meeting and to lists of past and future meetings. A meeting page (Figure 4) displays the meeting’s date and designated chairperson. It also shows a listing of cases added for discussion at this meeting, which is divided into cases to be discussed for the first time and those to be followed up from previous meetings. For each case in the list, its owner and and a descriptive case title are provided, as well as a link to the case presentation page.

**Figure 5:**
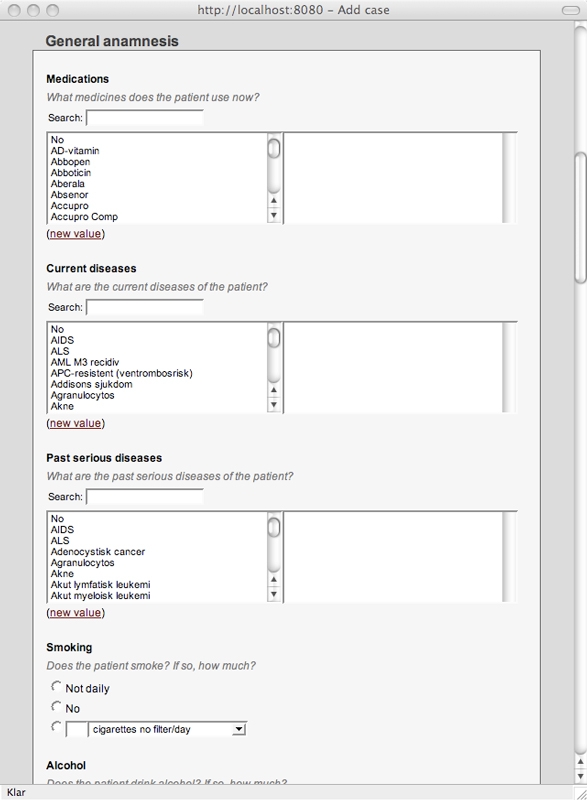
A form for adding a new case based on a user-defined consultation template

From the cases subheading, a user can add a case, display a listing of all cases, and access a free-text search of all cases. When wanting to add a case, the user is presented with a blank form (Figure 5), generated from a consultation template, the formalization of which is described in the next subsection. The form includes questions about, for example, current medications and tried treatments, and for each question, a list of allowed values is shown. If a value is missing, the user may enter it into the value list. The form also includes a free-text section. Images associated with the case are also submitted with this form. After adding a case, the user can assign it to be discussed at an upcoming meeting. A meeting for follow-up discussion can also be specified.

**Figure 6:**
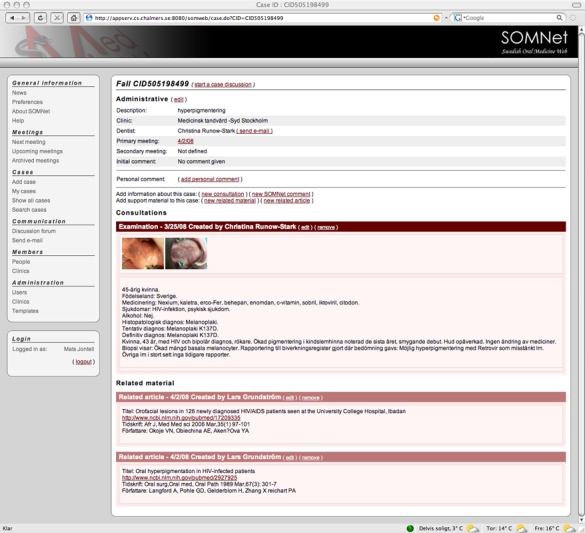
Case presentation page listing administrative data, consultation occasions with summarized case data and associated images, and related material

All submitted cases generate a case presentation page (Figure 6), which begins with administrative data: the case owner with affiliation, a short description provided by the owner, and any assigned meetings. Then, the case’s consultations are displayed. For each consultation, thumbnails of associated images are shown along with a presentation generated from the consultation data. From these thumbnails, a larger image browser can be accessed. Each case consists of a number of consultation occasions, and there are currently three different types of consultations: for initial case entry, for follow-up data, and for recording suggestions from meetings. These have separate entry forms with associated templates. All users can add follow-up consultations to cases, to make it possible for pathologists to add images and for users at the same clinic to share a case. Only the chairperson of a meeting can add notes to cases with suggestions from the meeting. All users can also add support material to cases, both in the form of articles and more generally related material such as images from a similar case. When entering an article, there is a facility for searching PubMed and automatically retrieving relevant article details.

A user can also choose to add private notes to cases. If a discussion thread exists for the case, it can be accessed from the presentation. If not, then clicking a link creates a new thread.

Browsing cases can be done via the meeting pages, pages listing cases of individual members, the list of all cases, or results of searching. From the “Members” subheading, users can access listings of all members and the clinics to which they belong, along with contact details.

A discussion forum listing all case discussions is located under the communication subheading. The users can also create threads not related to cases. Another communication facilitated by SOMWeb is reaching other clinicians by email. Messages can be sent directly from SOMWeb either from the communication subheading or to the case owner from a case presentation page.

It should be noted that SOMNet members have influence over the form of content and presentation of cases. First, case templates determining what information should be collected from submitted cases are the result of agreement between community members. As their needs change, they can update the templates themselves, using an editor in which they never interact with the underlying case representation. Second, summaries of cases are generated from stored data using community-defined presentation templates and natural language generation.

#### Semantic Web Technology Use in SOMWeb

Community aspects, such as users, meetings, cases, and news are modeled using OWL, and community data related to these are represented in RDF. Parts of the user descriptions make use of classes and properties from an external vocabulary called Friend of a Friend. All case data, that is all consultations, are stored in RDF.

**Figure 7:**
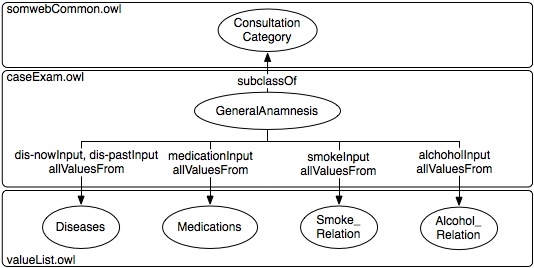
An example of a ConsultationCategory, GeneralAnamnesis, showing how OWL allValuesFrom restrictions are used to connect its inputs (properties) to classes of the value list ontology

The form used in entering each kind of consultation is generated from a community-defined template. The templates and associated value lists are represented in OWL (as described in [27]). Templates consist of categories with associated questions (also called inputs). When filling out a form, values for each input may be chosen from a specified class of the value list. Each template is stored in a different OWL file. Additionally, classes and properties common to all templates are defined in a separate OWL file. A template defines categories that can or need to be included in a consultation constructed from that template. For example, ConsultationCategory, a class common to all templates, is subclassed in an individual template by categories such as PatientData and MucosAnamnesis. Inputs are defined in the template, using OWL properties, along with what subclass of ConsultationCategory they are associated with and from what classes in the value list ontology values can be chosen. An input can also have properties with descriptions for when the input should be used and instructions to be shown when filling out the form. All clinical terms in the value list ontology (eg, Allergy) are represented as OWL classes, with their values as individuals (eg, PeanutAllergy). See Figure 7 for an example.

#### System Architecture

The SOMWeb system is a layered architecture with four main layers: view layer, session layer, model layer, and foundation layer. The view layer consists of Java server Pages using Expression Language constructs, including both custom tags and tags from the Java Standards Tag Library and various Apache struts tag libraries. Cascading Style Sheets are used for styling and layout of content. The components of the session layer deal with the current user session and transforming the application’s internal state into the presentation JavaBeans used by the server pages. The model layer handles the application’s internal state. This includes persistence classes, which create objects of the corresponding Java classes used by the system from RDF descriptions of users, meetings, cases, and news.

### Evaluation of SOMWeb

By May 2008, SOMWeb had 90 registered users located at 48 clinics; 93 added cases had been added and form the basis of a community repository of cases in oral medicine, and 18 meetings had utilized SOMWeb. Eleven users have submitted one case, five users have submitted two to four cases, four users have submitted five to six cases, four users have submitted seven to eight cases, and one user has submitted 19 cases.

#### Usability

All those interviewed stated that the SOMWeb system has improved the SOMNet collaboration. Several reasons were given: making case entry easier and less time consuming, prompting the supply of more uniform case data, enabling a collected view of a case over time, and providing more structure to SOMNet’s activities in general. Some also found that SOMWeb gave a greater sense of presence since it is possible to see in a clearer manner who other members are and who adds cases. Table 1 displays questionnaire responses comparing SOMWeb with the PowerPoint support.

**Table 1:** Users’ experience of SOMWeb’s functionality compared to the previous form of collaboration (These questions were answered by the 20 out of the 24 respondents who indicated that they had participated in SOMNet with PowerPoint support. Out of these 20, several did not answer all questions.)

Functionality	Rating Scale	Score
Using the case repository	Better/neutral/worse	14/4/0
Adding cases to the repository	Better/neutral/worse	13/2/0
Viewing old cases	Better/neutral/worse	15/2/0

When the PowerPoint presentations were used in the meetings, the users first went through the slides sequentially and then looked at specific slides as appropriate in the discussion. When using SOMWeb in meetings, users focus more on the images while listening to the presenter and rely on the textual case information for looking up facts as they need them. In SOMWeb, the cases are presented in the order in which they have been entered into the system, but the actual order of presentation is usually based on the preferences of the presenters.

Six of the nine interviewees had added cases. All of them found that it was easier to enter cases with the new system, yet only four found it very easy. The difference between these numbers indicates that there is some variation in opinion regarding case entry. Two respondents were concerned that the value lists had duplicate and misspelled entries, while two other respondents found the lists to be quite thorough and recognized that these will always contain some odd values. One respondent thought it took too much time to fill in the form and mainly used the free-text entry of the form. Another respondent found it difficult to select which data to enter for patients with a complicated clinical situation. Yet another respondent thought that some questions were missing from the form. Some users had trouble finding out how to submit a case for follow-up consultation.

Another identified problem is that images are sometimes loaded slowly when there is heavy system usage during meetings.

#### System Use

All interviewees use SOMWeb mainly in conjunction with meetings. All of them use the system a few days before the meeting or on the day of the meeting to go through cases and form their own opinion about the case. One person indicated that the personal comments in the system were used to remember these opinions. Another person used these comments to record notes during the meeting.

Though not explicitly asked of all interviewees, five reported that they had logged in to browse previous cases. One of these replied that this had never been done for the previous PowerPoint-based approach. Three interviewees had used the simple free-text search functionality but found that it needed improvement. Two others replied that the search had not been used since they found that the number of cases in the system did not yet warrant a need for searching.

#### Impact on Chairpersonship and Recording of Meeting Decisions

Included in the SOMWeb system is the possibility for the chairperson to add a meeting consultation to the discussed cases, where the group’s recommendations are entered. The idea is that after the meeting, the chairperson goes through any notes taken and adds relevant parts to each case. However, after about a year of use we found that this functionality was underused, partially because of a lack of time for the person chairing all meetings during this year. With SOMWeb also came the functionality of assigning a chairperson for each meeting. In June 2007, it was decided that the chair should rotate, partially because of the problem noted above. Since this decision, meeting preparations are more thorough and notes have been added for most cases (at five out of eight meetings since the start of rotation). For the meetings where notes were not added, reasons were found to be lack of knowledge that this should be done and lack of time. The instructions for the chairperson have been improved to alleviate this.

#### Impact on Use of External Evidence

In SOMWeb, article references can be added to all cases in a structured manner. This functionality was not included initially, and prior to this, articles were added as part of the chairperson’s notes. The news page has also been used to communicate articles of more general relevance. Though it was not part of the interview questions, four respondents indicated that they usually print and read the articles suggested. Only one of these has a research position and thus follows new publications independently of SOMWeb.

#### Impact on Collaboration Practices

The simple emailing facility of SOMWeb has also lead to more contact between the clinicians outside of meetings. Interviewees have found that this simple procedure saves them time in that they do not have to update their own address list.

When the SOMNet activities started more than 10 years ago, only four clinicians participated in the case discussions. In the beginning, PowerPoint presentations were distributed through email to less than 10 participants, all specialists in oral medicine. At that time, there were no passive members (ie, SOMNet comprised a small group of active clinicians who all participated in the discussions). The breakthrough came when clinical cases were distributed using SOMWeb. With little administrative effort, all members were able to access the website, and during the last 3 years, the number of members has increased tenfold. There is still a nucleus of five to 10 specialists who conduct most of the discussion. The more passive members are more inexperienced clinicians who learn from the main discussions and just occasionally ask questions. Holding more meetings has been suggested, though it has been brought up that each meeting might then get fewer participants. Another interviewee brought up the fact that the meetings feel stressed and that there is not time to discuss each case thoroughly. Dealing with this by limiting the number of cases has been discussed, but there has been no decision regarding who should decide what should be an adequate number of cases and how to choose between them.

## Discussion

Semantic Web technologies have been used for formalizing cases, examinations, and user data. As of May 2008, there were 90 registered users, 93 cases had been added to SOMWeb, forming a community repository of cases, and 18 meetings utilized the system. The introduction of SOMWeb has improved the structure of the meetings and the discussions that occur during the meetings, and a tenfold increase in the number of participants has been observed. Users have been found to submit cases to seek advice on diagnosis or treatment, to show an unusual case, or to create discussion. Identified barriers to submitting cases were lack of time, concern about whether the case was interesting enough, and showing gaps in one’s own knowledge. The provision in SOMWeb for assigning different chairpersons changed the collaboration in that responsibility for meeting preparations is now rotated.

### Comparison With Prior Work

#### Web-Based Systems for Clinical Practice and Research

Fearn et al present the Caisis system as a “web-based system for integrating clinical practice and research” [28]. There are several similarities between Caisis and SOMWeb. Both are based on a separation between data entry and data presentation, recognizing that these are in essence two different activities. Both adopt a user-centered approach with active involvement from clinicians. Finally, both Caisis and SOMWeb can be said to be structured around formalized patient histories (ie, cases). However, Caisis lacks SOMWeb’s foundation in Semantic Web technologies. A lesson learned from the Caisis project is that “as the system becomes more complex and feature-rich with each iteration, the learning curve becomes higher.” This problem is explicitly addressed in SOMWeb by using a “multi-layered” design for the user interface.

Based on Semantic Web technologies, the SWAN application aims at providing Alzheimer disease researchers with “an effective, integrated scientific knowledge infrastructure” [29]. The SWAN ontology and the SWAN information management tool are used for representing the different steps in a scientific discovery process and keeping track of hypotheses, with supporting evidence, research documents, clinical tests, and results in the form of data and publications. As far as we can tell, SWAN uses RDF only, not OWL. In terms of what is represented and handled by the system, SOMWeb and SWAN complement each other, and it would be interesting to try to use the SWAN ontology in the modeling of external evidence and see how it supports a case in SOMWeb.

Vega et al present “a cooperative working environment for sharing clinical experience over the Internet” [30]. Although focusing on image data, the presented solution is very similar to SOMWeb in terms of objectives and in focusing on “clinical sessions,” which in purpose and structure correspond to our “meetings.” However, the cases presented in clinical sessions are not formalized to the extent our cases are, and Semantic Web technologies are not used. As oral medicine is a discipline that is very much centered on images of the oral mucosa, the addition of the functionality presented by Vega et al for manipulating, annotating, and discussing images in real time to SOMWeb is currently being investigated.

Schleyer et al use an oral cancer center as an example of a biomedical research collaboratory [3]. In contrast to the collaborative work conducted within SOMNet, the functionality used by the researchers in the oral cancer center was more focused on distributed data analysis and preparation of publications than on “conversation over shared data, including, for example, images.” As is noted below, this is a shift in focus that we expect to see within oral medicine in the near future. In contrast to our approach, that of Schleyer et al is based on “off-the-shelf tools.” Without diminishing the advantages of this approach, it can be interesting to discuss its drawbacks in terms of SOMWeb: the problem of being dependent on adequate IT support is somewhat handled in SOMWeb by minimizing the need for IT and computer science experts by adopting end-user control. Problems associated with “poor interface design,” tools not being “well matched to tasks and technical progress,” and “how to integrate these tools into routine scientific practice” are explicitly addressed and handled in SOMWeb.

#### Communities of Practice

Wenger et al [14] suggest the need to design for evolution in supporting CoP. In the case of SOMNet, this is very much the case: they began with a simple technical solution, which has successively become more advanced and adjusted to their work processes. One can argue that it was necessary for the users to get used to the system functionality before they identified the need for new features (compare with [31]).

Inviting different levels of participation is another important principle in supporting CoP, and Wenger et al [14] propose that participants of a CoP can often be divided into core, active, and peripheral groups. This reflects the observation that, while it is hoped that all members participate equally, this is not a realistic expectation since different members participate in a CoP for different reasons. The core group consists of members that take on leadership roles and set the agenda for the group. The active members are regular participants in CoP events and sometimes participate in discussions, but not with the intensity or regularity of the core group. A large portion of the participants often belongs to the last group, which mostly observes interactions between core and active members. Reasons for not participating may be that they do not believe that their observations are valuable enough or that they do not have enough time. Wenger and colleagues [14] hold that these periphery activities are a very important part of CoP. Further, these peripheral members are not as passive as they might seem. They take in what is said and may bring it up in private conversations.

The different levels of member participation are clearly discernable in SOMNet. The core members chair meetings, contribute most cases, and are very involved in the discussions. The group of active users participates in most meetings, sometimes contributes comments and provides some cases. Finally, there is a large group of peripheral members who have not added cases and rarely or never make comments. As was noted previously, the number of peripheral participants has increased with the introduction of the SOMWeb system, and this has enabled the spread of oral medicine expertise beyond the core and active groups.

A third principle of Wenger et al [14] that can be seen at work in SOMNet is the community rhythm. The most prevalent rhythm is the monthly teleconferences. These affect when cases are entered and when members log in to the system. A system in which members added cases with a request for advice and in which other members could reply whenever would probably not work in this case. This conclusion is supported by the observations made by Moehr et al [31].

#### Multidisciplinary Medical Team Meetings

A SOMNet meeting can be seen as an instance of a multidisciplinary medical team meeting [32], where the team members meet to review patient cases, establish a diagnosis, and decide on the most appropriate treatment plan for the patient. The processes associated with a multidisciplinary medical team meeting system are pre-meeting activities; case presentation and discussion, including negotiation and reinterpretation of findings; deciding on the diagnosis and treatment; recording of the outcome; and postmeeting activities. As we have shown, a SOMNet meeting contains the same set of processes. It would be interesting to see how this structure could aid in the design of future versions of SOMWeb, adding possibilities for cuing chairpersons and participants in the discussions and securing that decisions being made are supported by relevant external evidence.

### Impact on Communication and Collaboration in SOMNet

In oral medicine, there is an ongoing discussion on how the discipline should move from an eminence to an evidence-based approach. The major hindrance to this amendment is the traditional manner of conducting clinical work. There is virtually no support to merge individual proficiencies with external knowledge. This barrier is particularly obvious between academic institutions and care providers serving different public health organizations. In this perspective, SOMWeb serves as an example of an expedient method to harmonize evidence-based knowledge. Apart from probably saving both time and effort [33], it is obvious that less experienced clinicians are learning from both submitting their own cases and from participating in discussions of cases presented by more experienced colleagues, who often practise at an academic institution. The opportunity within SOMWeb to agree on various treatment modalities and to evaluate the outcome of these suggested therapies are cornerstones of the learning process. Most likely, this exchange of case-related information will be followed by a demand for a more structured compilation of data of various disorders related to oral medicine, probably in the form of national registers. This movement is supported by SOMS, which has adopted SOMWeb as the national website for continuing education.

The main difference between SOMWeb and other similar initiatives for distance consultations within oral medicine is that, in latter systems, the clinical information is only shared between the specialist and the general practitioner. No efforts are made to save the data systematically for further use and comparison with similar cases. Furthermore, there is no follow-up of suggested treatment strategies, which will hamper the learning process. SOMWeb also brings in knowledge from external sources (eg, scientific papers). Thus, SOMNet internal experiences will be integrated with best available knowledge to the benefit of a single case, thereby contributing to a more evidence-based oral medicine.

One change that the SOMWeb system has provided is that SOMNet members are now more visible to each other via lists of members and what clinic they work at. Another important change is that the chairpersonship now rotates among core and active members. This has several benefits, such as reducing pressure on the original chairperson, which means that notes about the cases from the meeting are more consistently entered. It also means that more members feel involved in the work of SOMNet and that knowledge of how this work is carried out is spread to more people. Since it amounts to more external evidence, such as article references, being added, those clinicians less experienced in searching and using literature get more such exposure.

### Semantic Web Technologies in Practice

We have demonstrated the use of Semantic Web technologies to represent community and case data in an online community, where OWL could be used to address most but not all requirements for our knowledge model.

There are benefits of using formalized knowledge modeling for elucidating key concepts, and OWL has become widely used in this area. In addition, using OWL and RDF has made it easy to update the community model as the need has arisen. However, OWL is still evolving, and best practices have emerged while SOMWeb has been developed. We found a lack of guidance for several design choices and for development of OWL ontologies at different levels of sophistication.

A prospective benefit of using ontologies is the ability to reuse external sources, but we have not able to do this to the expected extent. Partially, this is due to the general lack of available ontologies in OWL. At a more foundational level, one can discuss to what extent ontologies can be readily reused since they are often developed with a certain purpose in mind. Some of the proposed benefits of Semantic Web technologies come from being able to share data with a larger audience. However, in the case of SOMWeb, such data sharing is not advisable given the nature of our data. Thus, we were not able to test the scalability of Semantic Web technologies in distributed systems.

### Conclusions

We have shown how an online Semantic Web-based CoP, SOMWeb, can successfully be developed and brought into daily clinical practice. In contrast to most work on CoP in the medical domain, SOMWeb aims at supporting activities related to both clinical practice and research within a distributed medical community, as exemplified by the SOMNet. Based on a firm foundation in knowledge representation and management, where OWL and RDF are used for representing community data and oral medicine knowledge, and on studies of collaboration and communication within SOMNet, functionality for Web-based distributed meetings has been developed iteratively, in close cooperation with the clinicians.

Studies and analysis of the use of SOMWeb show that it is beneficial for individual clinicians as well as for the SOMNet community. The introduction of SOMWeb has improved the structure of meetings and the discussions that take place, which constitute the core activities of SOMNet. Since the introduction of SOMWeb, there has been a tenfold increase in the number of meeting participants. SOMWeb provides an opportunity for its members to share high quality clinical practice knowledge as well as external evidence related to complex oral medicine cases, thereby contributing to a more evidence-based oral medicine.

As an example of an interdisciplinary team that can successfully address and solve complex research problems within the dental informatics domain [34], SOMWeb is the result of more than 10 years of collaboration between medical practitioners and researchers, computer scientists, and researchers within interaction design. This is probably the main success factor of the reported work. From the start, the composition of the development team included members acting as a “bridge” between the clinicians and the researchers, ensuring that the results of the latter are of real use and are adopted into practice by the former. A distinguishing feature of SOMWeb is the delegation of control of fundamental parts of the system to the end users. This means that the clinicians themselves have been able to adopt the system to their specific needs, requiring little interaction with computer specialists, contributing to the overall acceptance of the system. As an application of Semantic Web technologies, SOMWeb constitutes a sought-for experience report to the Semantic Web research community.

Within CoP, the importance of a champion is stressed. The champion is an authority within the domain in question, the driving force behind the work within the CoP, a precursor within the domain in terms of CoP-related technologies and tools, and the guiding example that others will follow. In the case of SOMWeb, a champion in this sense exists, together with a dedicated group of core users who are prepared to try out new ideas and solutions. In addition, SOMWeb has been designed to be aligned with the rhythm of the collaboration within SOMNet (ie, to use the SOMNet meetings as the basis for SOMWeb).

### Future Work

The overall aim of our research is to better understand collaboration and interaction among clinicians in order to improve IT tools that support evidence-based medicine.

In the short term, this translates to (1) continued study of the collaboration and communication within SOMNet and the use of the SOMWeb system; (2) further usage of the Semantic Web-based foundation, by using the domain ontology and reasoning (eg, to inform the browsing of cases) and by adding user and organizational ontologies; (3) adding functionality for real-time annotation of images during meetings; and (4) making the transition to Internet-based telephone services.

In the longer term, since cooperative care and knowledge sharing and dissemination are fundamental parts of evidence-based care in any medical discipline, developing SOMWeb into a general tool that builds online CoPs for other medical disciplines is an interesting prospect.

## Abbreviations

API: application programming interface
CoP: community of practice
CSCW: computer-supported cooperative work
HTML: HyperText Markup Language
IT: information technology
OWL: Web Ontology Language
RDF: Resource Description Framework
SOMS: Swedish Oral Medicine Society
SOMNet: Swedish Oral Medicine Network
